# A new thermography using inelastic scattering analysis of wavelength-resolved neutron transmission imaging

**DOI:** 10.1038/s41598-023-27857-0

**Published:** 2023-01-13

**Authors:** Hirotaka Sato, Mana Miyoshi, Ranggi Sahmura Ramadhan, Winfried Kockelmann, Takashi Kamiyama

**Affiliations:** 1 Division of Quantum Science and Engineering, Graduate School of Engineering, Hokkaido University, Kita-13 Nishi-8, Kita-ku, Sapporo, 060-8628 Japan; 2 Solid Mechanics Research Group, Department of Mechanical Engineering, University of Bristol, Bristol, BS8 1TR UK; 3 ISIS Neutron and Muon Facility, Rutherford Appleton Laboratory, Science and Technology Facilities Council, Didcot, OX11 0QX UK

**Keywords:** Techniques and instrumentation, Applied physics, Nuclear physics, Techniques and instrumentation

## Abstract

Thermography using energy-dependent neutron transmission imaging can non-invasively and non-destructively visualize a real-space distribution of interior temperatures of a material in a container. Previously, resonance absorption broadening analysis and Bragg-edge shift analysis using energy-resolved neutron transmission have been developed, however some issues remain, e.g., imaging efficiency, substance limitation and temperature sensitivity. For this reason, we propose a new neutron thermography using the temperature dependence of inelastic scattering of cold neutrons. This method has some advantages, for example, the imaging efficiency is high because cold neutrons are measured with moderate wavelength resolution, and light elements can be analysed in principle. We investigated the feasibility of this new neutron thermography at pulsed neutron time-of-flight imaging instruments at ISIS in the United Kingdom and HUNS in Japan. A Rietveld-type transmission spectrum analysis program (RITS) was employed to refine temperature and atomic displacement parameters from the inelastic scattering cross-section analysis. Finally, we demonstrated interior thermography of an α-Fe sample of 10 mm thickness inside a vacuum chamber by using a neutron time-of-flight imaging detector at the compact accelerator-driven pulsed neutron source HUNS.

## Introduction

Neutron thermography using energy-dependent neutron transmission imaging is expected to be a new remote sensing method that can measure a spatial distribution of interior temperatures over a large substance non-invasively and non-destructively. So far, epithermal neutron resonance absorption thermometry/thermography techniques using the nuclear Doppler effect have been developed as the first type of neutron thermometry using energy-resolved neutron imaging^1–7^. This method has some challenges insofar that energy-resolved epithermal neutron absorption spectroscopy is of low statistics in terms of neutron counts because the time-of-flight (TOF) method requires fine TOF channel widths in the range of nanoseconds. Detection efficiency for epithermal neutrons is lower than that for slow neutrons due to the 1/*v* law of neutron absorption. Temperature measurements via resonance analysis are impossible for some nuclides that do not show separable neutron-nucleus resonances. On the other hand, Bragg-edge neutron transmission using thermal or cold neutron TOF analysis can also be considered for neutron thermometry^8–11^. This method uses the thermal expansion of the crystal lattice and analyses the Bragg-edge wavelength shifts which reflect lattice parameters. However, the Bragg-edge shift evaluation requires high neutron wavelength resolution better than 1% and TOF analysis for a considerable range of tens of microseconds. Moreover, experimental imaging efficiency is low, and the method is limited to crystalline materials with Bragg-edges in a neutron transmission spectrum.

We developed a new neutron thermography using TOF analysis of cold neutrons as the third type of neutron thermometry using energy-dependent neutron transmission imaging. This new neutron thermography is based on the inelastic scattering analysis of cold neutrons^12^ which have lower energies, i.e., longer wavelengths, than resonance-captured and Bragg diffracted neutrons. For cold neutrons, the detection efficiency is enhanced, and a profile analysis of inelastically scattered neutrons does not require high wavelength resolution and fine TOF analysis, the latter of which are necessary requisites for the profile analysis of Bragg-edges and resonance absorption peaks. For these reasons, this new neutron thermography can be performed at relatively low intensity neutron facilities such as compact accelerator-driven pulsed neutron sources^13,14^ and relatively low energy resolution neutron facilities such as energy-selective neutron imaging instruments which do not use the TOF method^15–17^. One of the advantages of this new thermometry is that it can be applied to a range of materials and in the absence of neutron resonance absorption peaks, in principle.

The first aim of the present study was to investigate the temperature dependence of the inelastic scattering component in a neutron transmission spectrum. Here, we present data on α-iron, in order to demonstrate that inelastic neutron scattering analysis can provide temperature information, although other materials were explored or considered for follow-up studies. The second aim was to demonstrate a thermography in a container by using the temperature dependence of the inelastic scattering component in a neutron transmission spectrum. This methodology impacts on the application prospects of wavelength-resolved neutron transmission analysis of inelastic neutron scattering, in order to address various problems relating to a temperature mapping inside industrial products in the area of thermal and energy engineering.

In the second section of this paper, the basic principle of thermometry using inelastic neutron scattering in a neutron transmission setup is described. This is based on the slow neutron total cross-section analysis code called Rietveld Imaging of Transmission Spectra (RITS)^12,18–21^ which has been widely used for data analysis of Bragg-edge neutron transmission imaging. In the third section, we report experimental setups for measurements of temperature-dependent neutron transmission spectra and total cross-sections, including spatial-resolved measurements performed at the neutron TOF imaging instrument IMAT at the ISIS Facility, Rutherford Appleton Laboratory of the Science and Technology Facilities Council (STFC), United Kingdom^22^, and on a compact accelerator-driven pulsed neutron source, the Hokkaido University Neutron Source (HUNS) in Japan^13,14^. In the fourth section, we firstly discuss differences of temperature dependencies between calculated and experimental neutron total cross-sections. Then, the correction of calculation models of neutron total cross-sections for fitting the experimental data is reported. Finally, we report and evaluate the results using this new neutron thermometry measured within 2 h by using the compact accelerator-driven pulsed neutron source HUNS.

## Principle

In this section, calculation models for the temperature analysis of cold neutron transmission spectra and total cross-sections used in RITS^12,18–21^ are explained in detail. RITS can refine not only temperature *T* but also two atomic displacement parameters, *B*_iso_ and *φ*_1_*φ*_3_, and we note that these parameters are related to atomic dynamics. Finally, it is shown that the inelastic neutron scattering cross-section is sensitive to temperature.

### Neutron transmission spectrum and total cross-section

Here, it is considered that a sample is a polycrystalline material composed of a single element. This restriction does not limit the applicability to multi-atom compounds. The neutron transmission spectrum of a sample, *Tr*(*λ*), is experimentally measured through the ratio of the on-sample neutron spectrum, *I*(*λ*), to that of the off-sample beam, *I*_0_(*λ*), as follows:

1 $$Tr\left(\lambda \right)=\frac{I\left(\lambda \right)-BG\left(\lambda \right)}{{I}_{0}\left(\lambda \right)-BG\left(\lambda \right)},$$

where *λ* is the neutron wavelength derived from the TOF method, and *BG* is environmental background registered by a neutron detector. The environmental background sources include scattered neutrons from the sample itself but also from sample environment, detector, neutron beam dump and walls of the neutron irradiation room, as well as gamma-ray background. In our experiment, *BG* is assumed small (*BG* ~ 0) compared to the transmitted component because of low counts on a neutron detector in a neutron beam-collimated setup and because of a small solid angle for detecting scattered neutrons from sample and sample environment. The neutron transmission spectrum can be spatially resolved in a sample by using a neutron TOF-imaging detector.

The neutron transmission spectrum, *Tr*(*λ*), is expressed by the following equation:

2 $$Tr\left(\lambda \right)=\mathrm{exp}\left(-{\sigma }_{\mathrm{tot}}(\lambda )\rho t\right),$$

where *σ*_tot_(*λ*) is the neutron-nucleus microscopic total cross-section, *ρ* is the atomic number density, and *t* is the sample thickness. In this study, we derived *σ*_tot_(*λ*) from *Tr*(*λ*) by adjusting experimental *σ*_tot_(*λ*) values to match calculated *σ*_tot_(*λ*) values at a short wavelength around 0.1 nm because transmission data at this wavelength region are less sensitive to sample temperatures and potential background components. In particular, this procedure was necessary for a powder sample. *σ*_tot_(*λ*) is separated into four components, elastic coherent scattering, elastic incoherent scattering, inelastic scattering and absorption as follows:

3 $${\sigma }_{\mathrm{tot}}\left(\lambda \right)={\sigma }_{\mathrm{coh}}^{\mathrm{ela}}\left(\lambda \right)+{\sigma }_{\mathrm{incoh}}^{\mathrm{ela}}\left(\lambda \right)+{\sigma }^{\mathrm{inela}}\left(\lambda \right)+{\sigma }^{\mathrm{abs}}\left(\lambda \right).$$

The absorption cross-section of thermal/cold neutrons simply follows the 1/*v* law and does not depend on the atomic dynamics relating to temperature.

### Elastic coherent scattering

In the RITS code, the elastic coherent scattering cross-section is described as follows:

4 $${\sigma }_{\mathrm{coh}}^{\mathrm{ela}}\left(\lambda \right)=\frac{{\lambda }^{2}}{2{V}_{0}}\sum_{hkl}{\left|{F}_{hkl}\right|}^{2}{d}_{hkl}{P}_{hkl}(\lambda -2{d}_{hkl}){O}_{hkl}(\lambda ,2{d}_{hkl}){E}_{hkl}(\lambda ,2{d}_{hkl}).$$

  Here, *V*_0_ is the unit cell volume of the crystal lattice, *F*_*hkl*_ is the crystal structure factor, *d*_*hkl*_ is the crystal lattice plane spacing of {*hkl*} planes. *P*_*hkl*_(*λ*-2*d*_*hkl*_) is the Bragg-edge profile correction function due to instrumental resolution, micro-strain and crystallite size. In the RITS code, the Jorgensen-type function^23^ is used for *P*_*hkl*_(*λ*-2*d*_*hkl*_). *O*_*hkl*_(*λ*,2*d*_*hkl*_) is the preferred orientation correction function for crystallographic texture. In the RITS code, the March-Dollase function^24^ is used for *O*_*hkl*_(*λ*,2*d*_*hkl*_). *E*_*hkl*_(*λ*,2*d*_*hkl*_) is the primary extinction correction function. In the RITS code, the Sabine function^25^ is used for *E*_*hkl*_(*λ*,2*d*_*hkl*_).

The crystal structure factor, *F*_*hkl*_, is described by

5 $${F}_{hkl}=\sum_{n}{o}_{n}{b}_{n}\mathrm{exp}\left[2\pi i\left(h{x}_{n}+k{y}_{n}+l{z}_{n}\right)\right]\mathrm{exp}\left(-\frac{{B}_{\mathrm{iso}}(T){C}_{B}(T)}{4{d}_{hkl}^{2}}\right).$$

Here, *n* is the site in the lattice, *o* is the site occupancy, *b* is the scattering length, and (*x*, *y*, *z*) the fractional coordinates. $\mathrm{exp}\left(-\frac{{B}_{\mathrm{iso}}(T){C}_{B}(T)}{4{d}_{hkl}^{2}}\right)$ is the Debye–Waller factor which is traditionally known as exp(-*αTQ*^2^) where *T* is the temperature, *Q* is the neutron momentum transfer and *α* is a constant. We note that *C*_*B*_(*T*) does not exist in the traditional description of the Debye–Waller factor, as explained later. Thus, *B*_iso_ is proportional to *T* as *Q*^2^ dimensionally corresponds to 1/*d*^2^. The Debye–Waller factor depends on atomic dynamics relating to the temperature measurements in this study. The Debye–Waller factor becomes smaller with more intense atomic dynamics at higher temperatures. In addition, this factor does not depend on the neutron wavelength for the same {*hkl*} elastic coherent scattering cross-section.

The Debye–Waller factor includes the isotropic atomic displacement parameter, *B*_iso_(*T*), ignoring anisotropic displacements. *B*_iso_(*T*) can be calculated by the following equation^23,26^.

6 $${B}_{\mathrm{iso}}(T)=\frac{3{h}^{2}{\varphi }_{1}(T)}{M{k}_{\mathrm{B}}{\Theta }_{\mathrm{D}}}.$$

  Here, *h* is the Planck constant, *M* is the mass of nucleus, and *k*_B_ is the Boltzmann constant. Now, it is defined that

7 $$\Theta =\frac{T}{{\Theta }_{\mathrm{D}}},$$

where *T* is temperature, and *Θ*_D_ is the Debye temperature. By using Eq. (7),

8 $${\varphi }_{1}\left(T\right)=\frac{1}{2}+2\left[\Theta \mathrm{ln}\left(1-{e}^{-\frac{1}{\Theta }}\right)+{\Theta }^{2}\left(\frac{{\pi }^{2}}{6}-\sum_{j=1}^{\infty }\frac{1}{{e}^{\frac{j}{\Theta }}{j}^{2}}\right)\right],$$

according to Vogel^23^.

In the Debye–Waller factor, *C*_*B*_(*T*) is a correction factor for the atomic displacement parameter *B*_iso_(*T*), which is newly adopted and used in this study. *C*_*B*_(*T*) does not exist in the traditional description of the Debye–Waller factor. We propose the correction of the temperature dependence of *B*_iso_(*T*) which is proportional to the temperature in the traditional calculation model, by using the correction factor *C*_*B*_(*T*) as mentioned later. *C*_*B*_(*T*) is a non-dimensional parameter derived from the experimental data. In the RITS code, an atomic displacement parameter, *B*_iso_, can be refined by using the elastic coherent scattering profile fitting analysis, like for most Rietveld analysis codes for X-ray/neutron powder diffractometry.

### Elastic incoherent scattering and inelastic scattering

In the RITS code, the implementations for elastic incoherent and inelastic scattering cross-sections are the same as for CRIPO^27^ and BETMAn^23^. The inelastic scattering cross-section is described as follows^28,29^.

9 $${\sigma }^{\mathrm{inela}}\left(\lambda \right)={\sigma }_{\mathrm{coh}}^{\mathrm{inela}}\left(\lambda \right)+{\sigma }_{\mathrm{incoh}}^{\mathrm{inela}}\left(\lambda \right)=\left({\sigma }_{\mathrm{coh}}+{\sigma }_{\mathrm{incoh}}\right){S}_{\mathrm{incoh}}^{\mathrm{inela}}\left(\lambda \right),$$

and

10 $${S}_{\mathrm{incoh}}^{\mathrm{inela}}\left(\lambda \right)={S}_{\mathrm{incoh}}^{\mathrm{tot}}\left(\lambda \right)-{S}_{\mathrm{incoh}}^{\mathrm{ela}}\left(\lambda \right).$$

According to Granada^26^,

11 $${\sigma }_{\mathrm{incoh}}^{\mathrm{ela}}\left(\lambda \right)={\sigma }_{\mathrm{incoh}}{S}_{\mathrm{incoh}}^{\mathrm{ela}}\left(\lambda \right)={\sigma }_{\mathrm{incoh}}\frac{{\lambda }^{2}}{2{B}_{\mathrm{iso}}(T){C}_{B}(T)}\left[1-\mathrm{exp}\left(-\frac{2{B}_{\mathrm{iso}}(T){C}_{B}(T)}{{\lambda }^{2}}\right)\right].$$

Then, according to Placzek^30^, Granada^26^ and Vogel^23^,

12 $${S}_{\mathrm{incoh}}^{\mathrm{tot}}\left(\lambda \right)={\left(\frac{A}{A+1}\right)}^{2}\left(1+\frac{9{\varphi }_{1}\left(T\right){\varphi }_{3}\left(T\right){C}_{\varphi }(T){\lambda }^{2}}{2{A}^{2}{B}_{\mathrm{iso}}(T){C}_{B}(T)}\right),$$

where

13 $$A=\frac{M}{m},$$

and

14 $${\varphi }_{3}\left(T\right)=\frac{1}{4}+2\left[\Theta \mathrm{ln}\left(1-{e}^{-\frac{1}{\Theta }}\right)+6{\Theta }^{2}\left\{\frac{{\pi }^{4}}{90}{\Theta }^{2}-\sum_{j=1}^{\infty }\frac{1}{{e}^{\frac{j}{\Theta }}{j}^{2}}\left[\frac{1}{2}+\frac{\Theta }{j}+{\left(\frac{\Theta }{j}\right)}^{2}\right]\right\}\right].$$

  Here, *m* is the static mass of a neutron. *C*_*φ*_(*T*) is the correction factor for the atomic displacement parameter *φ*_1_(*T*)*φ*_3_(*T*) as a function of the temperature, which is newly adopted in this study. In the RITS code, not only *B*_iso_ but also the other single atomic displacement parameter *φ*_1_*φ*_3_ in Eq. (12) is refinable by using the inelastic scattering profile fitting analysis, as is the case with BETMAn^23^.

### Calculated temperature dependence

**Figure 1:**
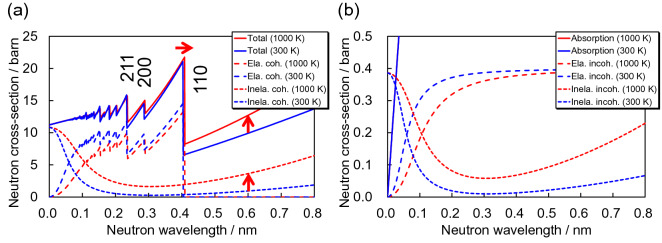
Neutron cross-sections of α-iron at 300 K and 1000 K, calculated by RITS. (**a**) Total cross-sections and coherent scattering cross-sections. (**b**) Absorption cross-sections and incoherent scattering cross-sections.

Figure 1 shows the total cross-section of α-Fe at temperatures of 300 K and 1000 K, by using the atomic displacement parameters *B*_iso_ and *φ*_1_*φ*_3_ calculated by the RITS code. The Debye temperature *Θ*_D_ was constant in our study, 470 K. In this data, the Bragg-edge wavelength shift due to thermal expansion of crystal lattice^31^ is considered. Temperature-dependent changes of scattering cross-sections are much more noticeable than the Bragg-edge wavelength shifts. At longer wavelengths than the Bragg cut-off, the total cross-section changes drastically with temperature. This phenomenon is mainly caused by change of inelastic coherent scattering in case of α-Fe.

This prediction is consistent with results of previous experimental work^10^, and we considered that this phenomenon can be utilised for high-sensitive temperature measurements of a sample. The RITS code was further developed for this purpose and used for thermography based on wavelength-resolved neutron transmission imaging experiments.

## Experimental

We measured temperature-dependent total cross-section data at pulsed neutron sources, ISIS and HUNS, using the TOF method. In this section, setups of each experiment are described.

### Measurement of temperature dependence of total cross-section of α-Fe at ISIS

The measurements of temperature-dependent total cross-sections were originally performed by previous work^10^, using the neutron TOF imaging instrument IMAT at ISIS, UK^22^. The neutron TOF-imaging detector was a microchannel plate (MCP) detector^32^. The pixel size was 55 μm × 55 μm, and the detection area was 28 mm × 28 mm. The IMAT beamline is connected to a coupled liquid hydrogen moderator on target station 2 of neutron pulse repetition rate of 10 Hz. A ‘pinhole’ collimator of 40 mm diameter at 46 m from the moderator was used; the collimation ratio at 10.5 m from the collimator, *L*/*D*, was about 250. The distance from the sample to the detector was 170 mm.

The sample was a powder of α-Fe, Goodfellows FE006020. The purity was 99.0%, and the particle size was smaller than 60 μm. The α-Fe powder was contained in a vanadium container of inner diameter 15 mm and 0.15 mm wall thickness. The sample was heated by the radiative element heater. To prevent oxidation and temperature instability of the sample, the sample set was contained in a vacuum furnace with a pressure of less than 0.1 Pa. The sample temperature was monitored by thermocouples, and the temperature instability was 2 K.

The selected temperatures were 293, 573, 673, 773, 873, 923, 973, 1023, 1073 and 1143 K. The neutron counts were summed over 274 × 274 pixels, namely, 15 mm × 15 mm of the MCP detector. The measurement time per temperature was 1.5 h for 9 temperatures, 2 h for 293 K, and 2.7 h for the off-sample data collection.

### Measurement of temperature dependence of spatial-resolved total cross-sections of α-Fe at HUNS

The temperature-dependent wavelength-resolved neutron transmission imaging experiments were performed at the Hokkaido University Neutron Source (HUNS) in Japan. The electron linear accelerator of Hokkaido University (Hokkaido LINAC) was replaced by a new one (Hokkaido LINAC-II) in 2018^33^. Hokkaido LINAC-II was operated as a pulsed photoneutron source. The electron energy was 32.5 MeV, the electron pulse width was 4 μs, the pulse repetition rate was 70 Hz, the time-averaged electron beam current was 70 μA, and the beam power was 2.3 kW. The neutron source and beamline were setup in high wavelength resolution mode for Bragg-edge neutron transmission imaging^14,34^. The neutron moderator was a decoupled-type polyethylene moderator at ambient temperature. A 3.65Qc supermirror guide tube of the 3.83 m length was installed. The neutron flight path length from the moderator to the detector was 6.277 m. For the reduction of background neutrons scattered from sample environment, a neutron grid collimator^34^ was coupled with a detector. The used neutron TOF-imaging detector was a gas electron multiplier (GEM) type^35^ produced by Bee Beans Technologies (BBT). The pixel size was 800 μm × 800 μm, and the detection area was 100 mm × 100 mm.

**Figure 2:**
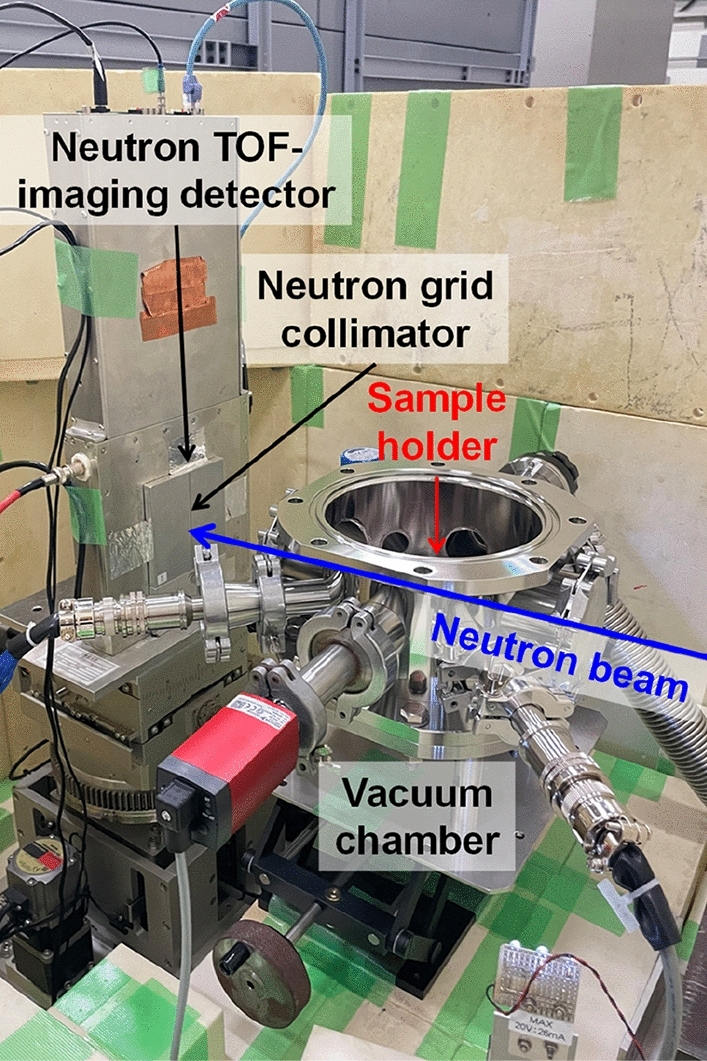
Setup of sample environment, neutron optical device and neutron TOF-imaging detector of the neutron thermography experiment performed at HUNS.

Figure 2 shows a photograph around sample and detector. The sample was an α-Fe plate, JIS-SS400, of thickness 10 mm and area 30 mm × 30 mm. The sample was heated by a Cu holder with cartridge heaters. The window size of the Cu holder was 20 mm × 20 mm, namely 25 × 25 pixels, corresponding to the region from which data were taken. The sample holder was set in a vacuum chamber for preventing oxidation and temperature instability of the sample. The vacuum level was less than 0.01 Pa. The sample temperature was monitored by thermocouples and the temperature instability was 3 K. The distance from the sample to the detector was 266 mm.

The selected temperatures were 294, 371, 465 and 569 K, lower than those selected for the ISIS experiments. The measurement times were 2 h for 294, 371, 465 K, 1.3 h for 569 K, and 4 h for the off-sample beam collection. The data measured at 569 K were not used for thermography demonstration due to the short measurement time.

## Correction of calculation model of neutron total cross-section for fitting to experimental data

In this section, we report differences of temperature dependencies between calculated and experimental neutron cross-sections through fitting using RITS. After that, corrections of temperature dependencies of atomic displacement parameters are discussed. *B*_iso_(*T*) × *C*_*B*_(*T*) was derived from the temperature-dependent elastic coherent scattering cross-sections. *φ*_1_*φ*_3_(*T*) × *C*_*φ*_(*T*) was derived from the temperature-dependent inelastic scattering cross-sections, including also *B*_iso_(*T*) × *C*_*B*_(*T*) determined from the elastic coherent scattering cross-sections.

### Comparison between calculated and experimental data

**Figure 3:**
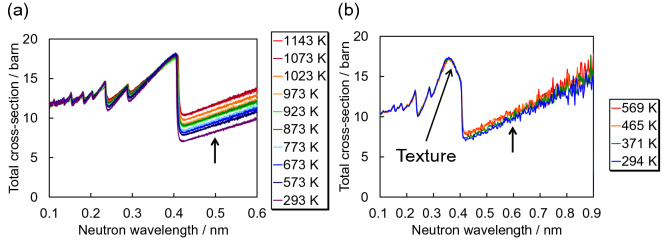
Temperature-dependent total cross-sections of α-iron measured (**a**) at ISIS from a powder sample and (**b**) at HUNS from an α-iron plate.

Figure 3 shows the temperature-dependent total cross-sections obtained from iron samples at ISIS and HUNS. Figure 3a represents the data at high temperatures from 573 to 1143 K including room temperature 293 K, and Fig. 3b represents the data at low temperatures from 371 to 569 K including room temperature 294 K. The elastic coherent scattering cross-sections of the α-Fe plate measured at HUNS indicate the presence of crystallographic texture. It is found that above the Bragg cut-off wavelength of α-Fe of about 0.4 nm the total cross-sections increase with increasing temperature. This can be attributed to the increase in inelastic scattering, as modelled in Fig. 1.

**Figure 4:**
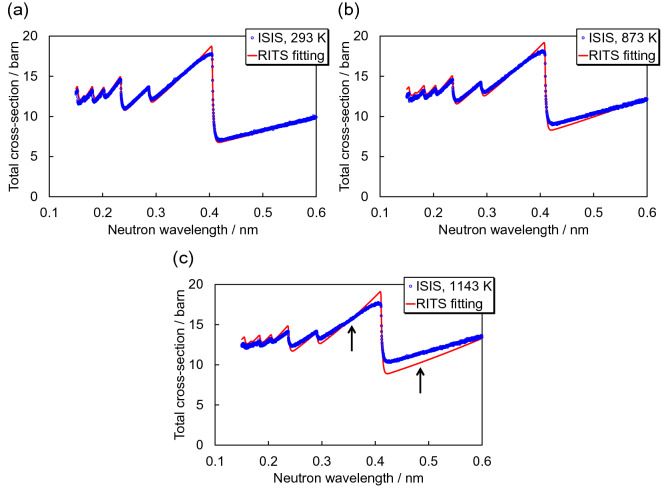
Comparison of experimental and calculated total cross-sections at temperatures of 293 K, 873 K and 1143 K.

For quantitative comparison of experimental and calculated data, temperature-dependent total cross-sections were calculated using RITS (Fig. 4). The calculated total cross-sections were tuned using the March-Dollase preferred orientation correction function and the Sabine primary extinction correction function. Whilst the calculated total cross-section at room temperature is in good agreement with the measured one, the calculated total cross-sections at high temperatures are not consistent with the measured ones, notably the wavelength-dependent slope of the inelastic scattering cross-section at longer wavelengths. The calculated curves exhibit steeper slopes than the measured ones. Also, the Bragg-edge heights between 0.3 and 0.4 nm were found to be different, as the RITS calculations predict slightly larger elastic coherent scattering cross-sections at the Bragg-edge positions.

Thus, the elastic coherent and inelastic scattering cross-sections calculated by RITS could not accurately reproduce the cross-sections measured in the high temperature experiments. For this reason, we implemented improvement of the calculation models representing temperature-dependent atomic dynamics.

The atomic displacement parameters, notably *B*_iso_ and *φ*_1_*φ*_3,_ are usually not based on calculations but are refined in a Rietveld analysis for X-ray/neutron powder diffractometry^36^. Therefore, we improved the temperature dependencies of *B*_iso_ and *φ*_1_*φ*_3_ by introducing correction factors, *C*_*B*_(*T*) and *C*_*φ*_(*T*). In the following sections, we discuss the details of elastic coherent and inelastic scattering cross-sections for deriving these correction factors.

### Correction of temperature dependence of atomic displacement parameter in the elastic coherent scattering cross-section

**Figure 5:**
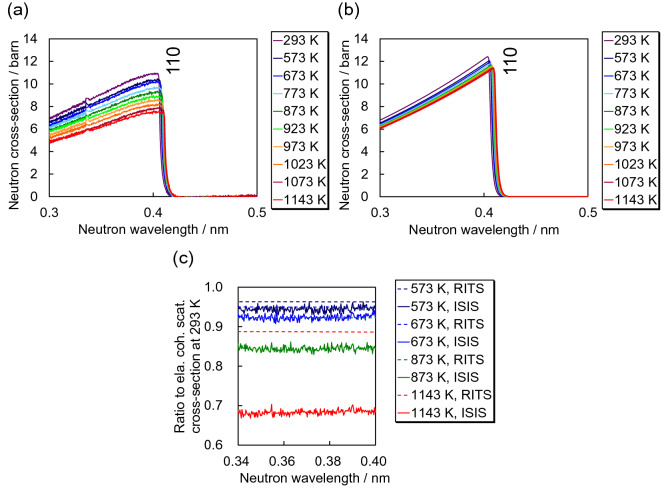
Extracted elastic coherent scattering cross-sections of data (**a**) measured at ISIS and (**b**) calculated by RITS. (**c**) Ratio to the elastic coherent scattering cross-section at 293 K.

In this section, the elastic coherent scattering cross-section is evaluated in detail. Firstly, we extracted only the elastic coherent scattering cross-sections of measured and calculated data. Figure 5 shows the results. The observed data were obtained at ISIS, and the calculated data were derived from RITS. The experimental elastic coherent scattering cross-sections were derived by subtraction of a linear function fitted to the total cross-section above the Bragg cut-off wavelength. Figure 5a and b show that the experimental elastic coherent scattering cross-section drastically reduces with increasing temperature, much more than the calculated cross-section which thus required a modification of the RITS model.

For further discussions, we evaluated the change of the wavelength dependence of the elastic coherent scattering cross-sections at each temperature. Figure 5c shows the ratio to the elastic coherent scattering cross-section at 293 K. In other words, elastic coherent scattering cross-sections at higher temperatures were normalized to the elastic coherent scattering cross-section at 293 K. Such processing was applied to experimental and calculated data separately. The ratio of the elastic coherent scattering cross-sections indicates a behaviour of the squared value of the Debye–Waller factor, *DWF*, as follows.

15 $$\frac{{\sigma }_{\mathrm{coh}}^{\mathrm{ela}}\left(\lambda \right)}{{\sigma }_{\mathrm{coh}\_293\mathrm{K}}^{\mathrm{ela}}\left(\lambda \right)}\approx \frac{{\left(DWF\right)}^{2}}{{\left({DWF}_{293\mathrm{K}}\right)}^{2}}.$$

This relation is reasonable if the lattice parameter changes, i.e., thermal expansion, are small. Figure 5c clearly shows that for higher temperatures the experimental ratios decrease much more than the calculated estimations, indicating much smaller Debye–Waller factors and larger *B*_iso_ values. Moreover, the cross-section ratios are almost wavelength independent, which is consistent with the expected behaviour of the Debye–Waller factor and *B*_iso_^37^ for the same lattice plane.

**Figure 6:**
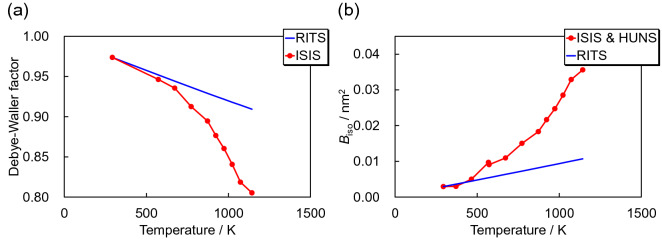
Temperature dependencies of (**a**) the Debye–Waller factor and (**b**) *B*_iso_ derived from the experimental and calculated data.

From Fig. 5c and Eq. (15), we derived the temperature dependence of the Debye–Waller factor as shown in Fig. 6a. Furthermore, from Fig. 6a, we derived Fig. 6b showing the temperature dependence of *B*_iso_, with the Debye–Waller factor approaching 1, and *B*_iso_ approaching 0 for the temperature approaching 0 K. In addition, the calculated *B*_iso_ is proportional to the temperature although this appears true only for the temperature approaching 0. These findings are reasonable in principle^37^, and consistent with the Debye–Waller factor defined in Eq. (5). Using the results of Fig. 6, modified models in RITS reproduce the experimental data at high temperatures. Finally, we used Fig. 6b to derive the correction function, *C*_*B*_(*T*), for *B*_iso_.

Incidentally, our results for elastic coherent scattering cross-sections are qualitatively and quantitatively consistent with results reported recently^11^ which were independently obtained.

### Correction of temperature dependence of atomic displacement parameter in the inelastic scattering cross-section

The inelastic scattering cross-section includes two atomic displacement parameters, *B*_iso_ and *φ*_1_*φ*_3_. Owing to Fig. 6b for the elastic coherent scattering cross-section, the temperature dependence of *B*_iso_ has been determined. By using these *B*_iso_ values, we evaluated the temperature-dependent *φ*_1_*φ*_3_ values from the temperature-dependent inelastic scattering cross-sections.

**Figure 7:**
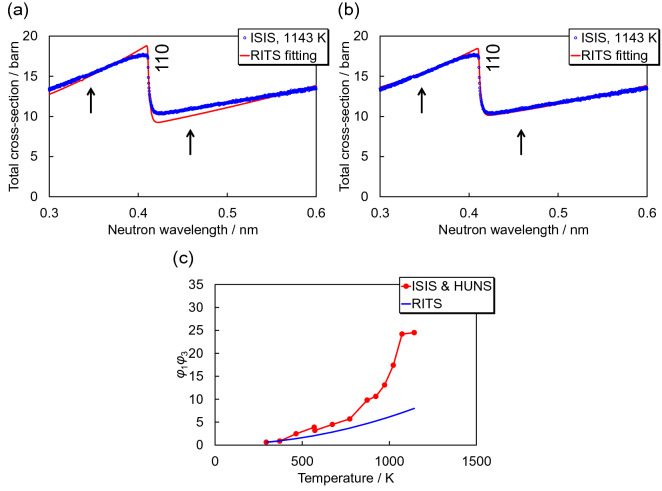
(**a**) Total cross-sections at 1143 K, measured at ISIS and calculated by RITS. (**b**) Total cross-section at 1143 K, measured at ISIS, with the calculated total cross-section using the correct/experimental *B*_iso_ and refined *φ*_1_*φ*_3_. Through refinement, *φ*_1_*φ*_3_ values were obtained from the experimental total cross-section. (**c**) Temperature dependence of *φ*_1_*φ*_3_ derived from the experimental data and the calculated data.

Figure 7a shows the total cross-section at 1143 K and calculated with the original RITS code. The reproduction is rather inaccurate as shown also in Fig. 4c. Figure 7b shows the corresponding comparison of cross-sections after refinement of *φ*_1_*φ*_3_ by using the experimental *B*_iso_ curve of Fig. 6b. For such a refinement, we finally derived the experimental *φ*_1_*φ*_3_ parameter product at each temperature (Fig. 7c). The experimental *φ*_1_*φ*_3_ values drastically increase, much more than calculated, uncorrected values as the sample temperature increases. Finally, we used Fig. 7c for the temperature-dependent correction function *C*_*φ*_(*T*) for *φ*_1_*φ*_3_, as well as Fig. 6b for *B*_iso_.

### Correction functions and their application to thermometry

**Figure 8:**
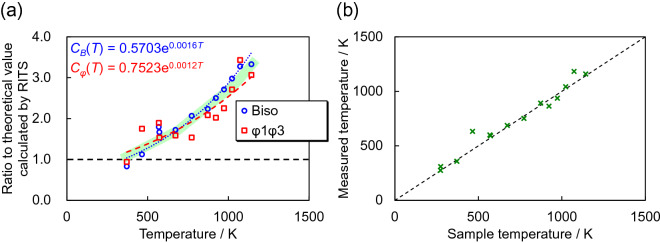
(**a**) Correction functions *C*_*B*_(*T*) for *B*_iso_(*T*) and *C*_*φ*_(*T*) for *φ*_1_*φ*_3_(*T*), derived from the ratio of experimental *B*_iso_(*T*) and *φ*_1_*φ*_3_(*T*) to calculated ones. (**b**) Temperature analysis results by fitting the total cross-section calculated by RITS with the correction functions *C*_*B*_(*T*) and *C*_*φ*_(*T*) to the experimental total cross-sections.

For determination of the correction functions *C*_*B*_(*T*) for *B*_iso_(*T*) and *C*_*φ*_(*T*) for *φ*_1_*φ*_3_(*T*), we calculated the ratio of experimental *B*_iso_(*T*) and *φ*_1_*φ*_3_(*T*) to calculated *B*_iso_(*T*) and *φ*_1_*φ*_3_(*T*), corresponding to *C*_*B*_(*T*) and *C*_*φ*_(*T*). Figure 8a shows the results. The experimental *B*_iso_(*T*) and *φ*_1_*φ*_3_(*T*) are several times larger than the calculated *B*_iso_(*T*) and *φ*_1_*φ*_3_(*T*). This indicates that thermography using an inelastic scattering analysis of wavelength-resolved neutron transmission data is more sensitive to the sample temperature, compared with the advance prediction as shown by Fig. 1. In addition, and interestingly, the temperature-dependent correction factors of *B*_iso_(*T*) and *φ*_1_*φ*_3_(*T*) approximately match quantitatively, although the correction functions *C*_*B*_(*T*) and *C*_*φ*_(*T*) were independently determined in our present study. We fitted the correction functions *C*_*B*_(*T*) and *C*_*φ*_(*T*) by using an exponential function (Fig. 8a), such that

16 $${C}_{B}\left(T\right)=0.5703{e}^{0.0016T}$$

and

17 $${C}_{\varphi }\left(T\right)=0.7523{e}^{0.0012T}$$

were determined as the correction functions for *B*_iso_(*T*) and *φ*_1_*φ*_3_(*T*).

Finally, we analysed the temperature-dependent total cross-section data measured at ISIS and HUNS by using RITS with the correction functions *C*_*B*_(*T*) and *C*_*φ*_(*T*). *T* was a refinable parameter while *Θ*_D_, *C*_*B*_(*T*) and *C*_*φ*_(*T*) were fixed parameter and functions. Figure 8b shows the results. The measured temperatures obtained from neutron transmission correlate with the sample temperatures, as expected, where the scatter of the measured temperatures is due to the fitted correction functions (Eqs. 16 and 17) representing the correction factors (Fig. 8a). Owing to the correction functions, reasonable temperature measurements as demonstrated in Fig. 8b are feasible, enabled by the inelastic scattering analysis of wavelength-resolved neutron transmission imaging.

In view of future work, we note remaining issues relating to the temperature dependence of the inelastic scattering cross-section. Firstly, this concerns confirmation whether the correction method for atomic displacement parameters (Fig. 8a) is generally applicable. In a follow-up study we have found that this relationship can also be applied to aluminium, however, the Debye temperatures of Al (428 K) and Fe (470 K) are close, and the universality of Fig. 8a should be further investigated using other substances. Furthermore, the Debye temperature is, in general, temperature dependent whilst Eqs. (6), (8) and (14) use single values. In addition, the inelastic scattering cross-section calculated by RITS is based on the incoherent approximation. Several approaches such as the quasi-harmonic approximation^38^ to include the inelastic coherent scattering, i.e., phonon dependencies on the neutron momentum transfer, have to be tested in the future because the incoherent approximation may be unsuitable for reconstruction of temperature. Thus, further improvements of theoretical functions in the RITS code are envisaged for future work for application of temperature mapping using inelastic neutron scattering analysis.

## Thermography demonstration

Finally, we demonstrated the new neutron thermography using spatial-resolved inelastic scattering analysis of an α-Fe plate of 10 mm thickness measured at HUNS. The neutron counts were analysed using a running-average of 3 × 3 pixels of the GEM detector with a step size of 800 μm.

**Figure 9:**
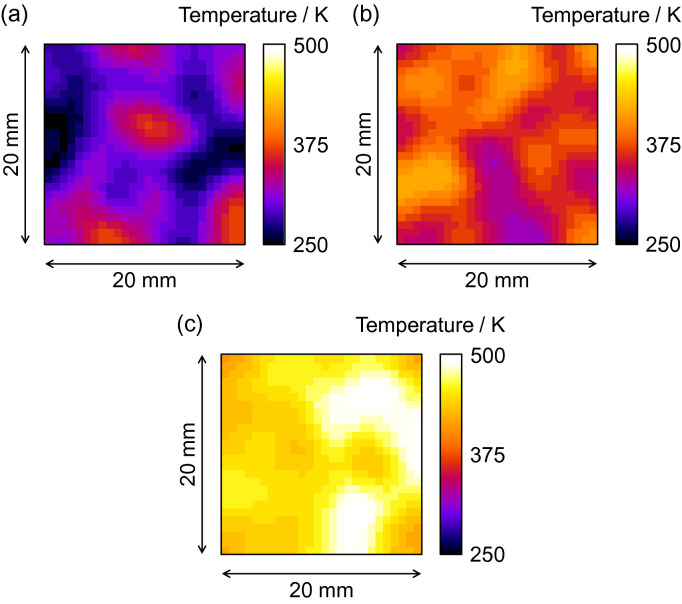
Interior, spatially resolved thermography of an α-Fe plate of 10 mm thickness inside a vacuum chamber by using inelastic neutron scattering analysis at HUNS. The sample temperatures, read with a thermocouple, were (**a**) 294 K, (**b**) 371 K and (**c**) 465 K while temperatures of 304 ± 27 K, 370 ± 24 K and 458 ± 20 K, respectively, were obtained from the pulsed neutron imaging experiment.

Figure 9 shows the result of spatially-resolved thermography on the α-Fe sample at temperatures of 294 K, 371 K and 465 K. Note that these temperature images could be obtained in 2 h at the compact accelerator-driven pulsed neutron source facility. It is visually confirmed that the temperatures evaluated from the inelastic scattering analysis increase as the sample temperature rises. The averages and standard deviations of evaluated temperatures were 304 ± 27 K, 370 ± 24 K and 458 ± 20 K, respectively. The accuracy evaluated from the averages was better than 10 K, and the precision from the standard deviations was smaller than 27 K. It is interesting to note that the precision becomes better at high temperatures as the temperature-sensitivity increases as shown in Fig. 8 (a). Thus, it was demonstrated that an inelastic scattering analysis of position-dependent total cross-section data measured by wavelength-resolved neutron transmission imaging is usable for thermography of a thick metal sample inside a container.

## Conclusions

We developed a new thermography by analysing inelastic scattering of wavelength-resolved neutron transmission images. In many cases, the change of the inelastic scattering cross-section is more sensitive to a change of sample temperature than the previous developed thermometry using Bragg-edge shifts. Whereas temperature mapping techniques using Bragg-edge and resonance absorption require sufficiently high wavelength resolution, inelastic scattering analysis can be performed with coarse wavelength resolution. The imaging efficiency of the new method is relatively high and light element compounds can be analysed in principle. The new neutron thermography method takes advantage of the functionality of the RITS code which is traditionally used to derive crystalline and microstructural properties from total cross-sections. Calculation models used in RITS predict experimental total cross-sections reasonably well, however not with sufficient accuracy for thermography at high temperatures, where the parameters of the Debye approximation (Eqs. (6), (8) and (14)) are not always well known. For this reason, we modified the calculation of temperature-dependencies of atomic displacement parameters *B*_iso_ and *φ*_1_*φ*_3,_ and determined appropriate correction functions which made total cross-section profile fitting and temperature analysis feasible. Finally, we demonstrated an interior thermography of an α-Fe sample of 10 mm thickness in a vacuum chamber at HUNS, a compact accelerator-driven pulsed neutron source. The measured temperature maps at 304 ± 27 K for a targeted temperature of 294 K, 370 ± 24 K for 371 K and 458 ± 20 K for 465 K, respectively, were measured in 2 h.

Thus, the first demonstration of a remote temperature sensing method using inelastic scattering analysis of wavelength-resolved neutron imaging data was successfully carried out. On the other hand, it remains to be confirmed that the proposed correction functions of the atomic displacement parameters are applicable to substances other than iron: for different materials, for temperature regimes where the Debye model is not a good approximation, and across phase transition temperatures. Alternatively, approaches such as the quasi-harmonic approximation^38^ used in the NCrystal code^39^ may be more suitable than the incoherent approximation for reconstruction of temperature dependencies of inelastic scattering cross-sections. Temperature tomography using inelastic scattering cross-section analysis is also a possible next step, expedited by high cold neutron fluxes at Mega-Watt spallation neutron sources which will allow measuring temperature projections in much shorter times and for a range of materials. Atomic dynamics science and thermal engineering applications will be potentially available at energy-dependent neutron imaging stations.

## Data Availability

The datasets used and/or analysed during the current study are available from the corresponding author on reasonable request.
